# First Report of *Phytopythium vexans* Causing Stem and Leaf Necrosis on Rubber Tree (*Hevea brasiliensis*): Biological Characteristics and Fungicide Sensitivity

**DOI:** 10.3390/plants15162419

**Published:** 2026-08-07

**Authors:** Zhiying Cai, Lili He, Liming Dai, Yuping Shi, Yixian Liu, Haipeng Su, Sipeng Jian, Hongjun Mu

**Affiliations:** Tropical Crop Diseases Research Group, Research Center of Plant Protection, Yunnan Key Laboratory of Sustainable Utilization Research on Rubber Tree, Yunnan Institute of Tropical Crops, Jinghong 666100, China; caizhiyingyn@163.com (Z.C.); hll3850@163.com (L.H.);

**Keywords:** fungicides, *Hevea brasiliensis*, identification, *Phytopythium vexans*

## Abstract

*Hevea brasiliensis* (Willd. ex A.Juss.) Müll.Arg., the world’s primary source of natural rubber, faces increasing threats from emerging diseases in tropical cultivation regions. During recent field surveys in Yunnan and Hainan provinces of China, previously uncharacterized brown, water-soaked necrotic lesions were observed on tender stems and leaves of rubber trees. This study aimed to identify the causal pathogen, characterize its biological traits, and evaluate candidate fungicides for disease management. The pathogen was isolated via tissue explant methods and identified through morphological observation combined with multigene phylogenetic analyses based on the internal transcribed spacer (*ITS*) region, large subunit (*LSU*) ribosomal DNA, and cytochrome *c* oxidase subunits I and II (*coxI* and *coxII*) sequences. Pathogenicity was rigorously confirmed by fulfilling Koch’s postulates on rubber tree seedlings. The isolates produced spherical to ovoid, papillate sporangia, and phylogenetic reconstruction strongly supported clustering them with *Phytopythium vexans*. The pathogen grew optimally at 16–31 °C, with growth ceasing at 35 °C. Fungicide sensitivity assays revealed that metalaxyl exhibited the strongest inhibitory activity against mycelial growth, with half-maximal effective concentration (EC_50_) values as low as 0.04 μg/mL. To our knowledge, this is the first report of *P. vexans* causing foliar and stem necrosis on *H. brasiliensis*. These findings provide a theoretical foundation for accurate disease diagnosis, epidemiological surveillance, and the development of effective chemical control strategies for rubber plantations.

## 1. Introduction

*Hevea brasiliensis* (Willd. ex A.Juss.) Muell.Arg., an evergreen tree belonging to the genus *Hevea* in the family Euphorbiaceae, is the primary source of natural rubber worldwide. Native to the Amazon rainforest in South America, it is now widely distributed across Asia, Africa, and the Americas, exhibiting a pantropical distribution pattern [1,2,3]. Among over 2500 rubber-producing plant species, *H. brasiliensis* is the only one capable of producing high-quality natural rubber with commercial value, accounting for more than 98% of the global natural rubber production [3]. The latex secreted by rubber trees contains *cis*-1,4-polyisoprene [4,5,6], proteins, phospholipids, and other components. The microstructural network formed by these components endows natural rubber with unique physicochemical properties, including efficient thermal dispersion, impact resistance, and tear resistance-properties that synthetic rubber has yet to fully replicate [7,8]. Natural rubber is an indispensable strategic raw material in the global industrial system. Its excellent elasticity, abrasion resistance, and tensile strength make it a key material for over 40,000 products [7,9], serving as a critical link between basic industries and advanced manufacturing.

Oomycetes are a distinctive group of eukaryotic microorganisms that morphologically resemble fungi but are phylogenetically distinct. Unlike true fungi, their cell walls contain cellulose, a feature that fundamentally distinguishes them from fungi. Many oomycete taxa pose significant threats to the health of both animals and plants [10]. Within this group, oomycetes are best known as plant pathogens, capable of causing various devastating plant diseases that severely impact agricultural productivity and ecosystem stability [11,12]. Among the pathogenic oomycetes, the genera *Pythium* and *Phytophthora* are the most prominent in terms of the damage they cause [12]. For instance, *Phytophthora infestans*, a representative species of the genus *Phytophthora*, triggered the Great Irish Potato Famine in the 19th century [13] and continues to cause billions of dollars in annual economic losses to global agriculture. On the Pacific coast of the United States, *Phytophthora ramorum*, the causal agent of sudden oak death, has led to widespread tree mortality, severely disrupting natural vegetation [14]. *Pythium* species, on the other hand, typically infect plant roots or seeds, causing various diseases. Together, these two genera not only cause drastic yield reductions (up to 20–40% in severe cases) in major crops such as potatoes, soybeans, and peppers, leading to substantial agricultural economic losses, but also persistently threaten the species diversity of natural ecosystems [15].

In rubber plantations, *Phytophthora* species are known to cause leaf fall and bark canker, leading to significant economic losses, particularly in humid tropical regions [16,17]. Leaf fall disease primarily affects leaves, tender branches, and rubber fruits. Infected young leaves develop dark green, water-soaked lesions that rapidly turn black and abscise. In mature leaves, infection begins at the base of the petiole; the exuded gel from the lesion turns from white to black, affected branches appear scorched and withered, and infected fruits rot, eventually drying into hard mummified fruits that remain attached to the branches. Bark canker disease damages the tapping panel of rubber trees. Newly cut panels first develop palisade-like black lines that penetrate deep into the xylem. Under humid conditions, white mildew grows on the lesions. Subcutaneous latex exudation causes the bark to burst; the xylem turns dark brown, and in severe cases, the bark over nearly half the trunk circumference may rot [18,19]. These diseases are now prevalent across all rubber-producing regions worldwide [20,21]. In China, high-incidence areas include Wanning and Danzhou in Hainan Province, as well as Xishuangbanna and Mengla in Yunnan Province, where hot and humid conditions prevail [22,23,24]. According to local agricultural statistics, in 2024 alone, the affected area in Xishuangbanna Prefecture, Yunnan Province, exceeded 33,300 hectares, resulting in economic losses of over 50 million CNY. The epidemiology of these diseases has been reported to be closely associated with climatic conditions in previous studies [25]. Continuous rainfall during the monsoon season, along with high humidity and cool conditions within rubber plantations, creates favorable conditions for pathogen spread. Typhoon damage and other physical injuries to trees further increase the risk of disease outbreaks. Moreover, pathogens carried by infected leaves and fruits can be washed by rainwater onto the tapping panel [26], establishing a “leaf fall-stem canker” chain of infection, which poses a dual threat to tree vigor and latex yield [17,27]. During a survey of rubber tree leaf fall disease, the research team isolated several colonies resembling *Phytopythium* sp. from blackish-brown, water-soaked necrotic tender stems and leaves of rubber trees, characterized by spherical oospores and white mycelia.

The genus *Phytopythium* is a relatively recently established lineage, phylogenetically intermediate between *Pythium* and *Phytophthora*, and shares morphological and biological characteristics with both genera [28,29]. Although these similarities exist, *Phytopythium* possesses several distinct morphological features that facilitate its identification. The genus currently comprises over 20 species, most of which are saprophytic; however, several species—including *Phytopythium litorale*, *P. helicoides*, *P. chamaehyphon*, and *P. vexans*—are highly pathogenic to plants [16,29,30,31,32,33].

Despite its broad host range and morphological resemblance to *Phytophthora*, *P. vexans* has never been reported on rubber trees. Indeed, all previously documented oomycete diseases of *H. brasiliensis* have been attributed exclusively to *Phytophthora* species, raising concerns that *Phytopythium* infections may have been overlooked or misidentified. To address this knowledge gap, three hypotheses were formulated: (1) the oomycete isolates recovered from diseased rubber stems and leaves belong to *P. vexans*, as determined by multi-gene phylogenetic analysis; (2) these isolates are primary pathogens capable of inducing typical symptoms on healthy rubber seedlings, fulfilling Koch’s postulates; and (3) metalaxyl, a phenylamide fungicide with specific anti-oomycete activity, exhibits superior inhibitory efficacy compared with fungicides targeting different pathways. Accordingly, this study aimed to: (i) verify the pathogenicity of the isolated *Phytopythium* sp. on rubber trees using Koch’s postulates; (ii) identify the pathogen through molecular and morphological characterization; and (iii) screen for fungicides with significant inhibitory effects on mycelial growth via in vitro plate assays.

## 2. Materials and Methods

### 2.1. Sample Collection

Disease surveys were conducted in the major rubber-producing regions of Yunnan and Hainan Provinces, China, from 2024 to 2025. A total of 45 symptomatic samples (35 leaf samples and 10 stem samples) were collected from five different plantations located in Jinghong City (21°55′ N, 100°48′ E, 580 m a.s.l.) and Mengla County (21°29′ N, 101°34′ E, 620 m a.s.l.) in Yunnan Province, and Wanning City (18°48′ N, 110°24′ E, 20 m a.s.l.) and Danzhou City (19°31′ N, 109°34′ E, 130 m a.s.l.) in Hainan Province. Fresh samples exhibiting localized blackening of leaves or stems were collected for pathogen isolation, and detailed geographic information was recorded for each sample.

### 2.2. Isolation and Purification

The pathogen was isolated using the tissue isolation method [32]. Tissue blocks measuring 0.5 cm × 0.5 cm were excised from the interface between diseased and healthy tissues of rubber tree organs. Under a clean bench, the tissue blocks were immersed in 75% ethanol for 3–5 min. After removal from the ethanol, the blocks were placed in sterile Petri dishes. Following evaporation of residual ethanol, the tissue blocks were transferred onto selective potato dextrose agar (PDA) medium supplemented with 100 μg/mL ampicillin, 100 μg/mL cephalosporin, and 20 μg/mL carbendazim. Four to five tissue blocks were placed per Petri dish and incubated in darkness at 28 °C for 2–3 days. After the emergence of white mycelia, the tips of aerial hyphae were picked and re-inoculated onto the same medium for purification. The purified strains were then numbered. From the 45 symptomatic samples, a total of five oomycete strains were obtained and purified, designated as HNJC71, HNJC73, HNJC87, HNJC89, and HNJC90. Strain HNJC71 was isolated from a diseased rubber leaf collected from Wanning City, Hainan Province (19°05′98′′ N, 109°79′84′′ E). Strain HNJC90 was isolated from a diseased rubber stem collected from Danzhou City, Hainan Province (19°03′16′′ N, 109°76′23′′ E). Based on preliminary phylogenetic analysis of the *ITS* region using all five isolates, strains HNJC71 and HNJC90 were selected as representative isolates for detailed morphological characterization, multi-gene phylogenetic analysis, and fungicide sensitivity testing. Agar blocks containing the mycelia were cut and placed into preservation tubes containing sterile distilled water and stored at 18 °C for subsequent use. This storage temperature follows the rye vial method for prolonged preservation of oomycete cultures, as described by Abad et al. [33], which allows storage at 18–20 °C for up to 2 years.

### 2.3. Pathogen Identification

Of the five isolates obtained, all were subjected to *ITS*-based molecular identification. Detailed morphological characterization, multi-gene phylogenetic analysis (*ITS*, *LSU* rDNA, *coxI*, and *coxII*), and biological characterization were performed on the two representative isolates HNJC71 and HNJC90.

#### 2.3.1. Morphological Observation and Biological Characterization

Morphological observation and biological characterization were performed on the two representative isolates HNJC71 and HNJC90. Following established methods [12,16,28,29], the morphological characteristics of each isolate were observed and recorded, including colony morphology, hyphal swelling structures, sporangia, chlamydospores, and the arrangement of oogonia and antheridia. A Zeiss Axio Imager A2m (Carl Zeiss AG, Oberkochen, Germany) microscope was used to observe and measure sporangial size at 40× magnification. For each isolate, more than 50 sporangia were randomly observed, and the experiment was repeated three times. Subsequently, the isolates were preliminarily identified by consulting the relevant literature [12,16,28,29,34].

To characterize colony morphology, the isolates were inoculated onto PDA, malt extract agar (MEA), V8 juice agar, and carrot agar (CA). The cultures were incubated in darkness at 25 °C for 3 days, after which colony morphology and color were recorded.

To determine the optimal growth temperature of the isolates, mycelial plugs (6 mm in diameter) were inoculated onto fresh PDA medium in 90 mm Petri dishes. The cultures were incubated at temperature gradients ranging from 10 °C to 35 °C at 3 °C intervals. A total of 10 temperature treatments were included. For each isolate, five replicate plates were used per temperature treatment. Colony diameters were measured and recorded daily. The entire experiment was repeated three times.

#### 2.3.2. Molecular Identification and Phylogenetic Analysis

Genomic DNA was extracted using the Biospin Fungal Genomic DNA Extraction Kit (Bioer Technology Co., Ltd., Hangzhou, China) according to the manufacturer’s instructions. Four genomic regions were amplified using primer pairs listed in Table 1: the internal transcribed spacer (*ITS*) region, large subunit (*LSU* rDNA), and cytochrome *c* oxidase subunits I and II (*coxI* and *coxII*). The four loci were selected based on their complementary evolutionary characteristics. The *ITS* region provides high interspecific variability for distinguishing closely related species, while the *LSU* rDNA is more conserved and offers reliable resolution at higher taxonomic levels. The mitochondrial *coxI* and *coxII* genes evolve more rapidly and provide independent phylogenetic signals, which are particularly valuable for resolving species complexes. Concatenated analysis of these four loci significantly increases phylogenetic resolution and statistical support compared with single-gene analyses and aligns with the current standard for oomycete identification as recommended by de Cock et al. [29]. Each 50 μL PCR reaction mixture consisted of 25 μL of Green PCR Master Mix, 17 μL of sterile deionized water, 4 μL of DNA template, and 2 μL each of forward and reverse primers. The thermal cycling protocol was: initial denaturation at 94 °C for 3 min; 35 cycles of 94 °C for 30 s, annealing at the temperatures listed above for 30 s, and 72 °C for 60 s; and a final extension at 70 °C for 10 min. The PCR products were electrophoresed on a 2% agarose gel at 150 V for 25 min. PCR products were sequenced by Kunming Tsingke Biotechnology Co., Ltd. (Kunming, China).

Firstly, sequence processing was performed using Sequencher v4.1.4 (Gene Code Corp., Ann Arbor, MI, USA) to assemble sequences, remove regions of ambiguous bases, and merge degenerate bases. Then, sequences were aligned with MAFFT v7.526 using the E-INS-i strategy [35] and manually verified in BioEdit v7.7.1 [36]. Each single-gene dataset was checked for topological incongruence; Maximum Likelihood (ML) analyses were conducted on every single-gene dataset. All single-gene alignments were then concatenated using Phyutility v2.2 [37], with any missing sequence data coded as gaps, to generate a final super-matrix of all four gene loci.

Phylogenetic trees were constructed using concatenated sequences via Bayesian inference (BI) and maximum likelihood (ML) methods. The best-fitting substitution model for each dataset was determined using MrModelTest 2.3 [38] under the Akaike Information Criterion (AIC). ML analysis of the combined dataset was conducted with RAxML 7.2.6 [39] applying the GTRGAMMAI model. Statistical support for internodes was assessed via non-parametric bootstrapping with 1000 replicates.

For BI analysis of the combined dataset, a partitioned mixed-model approach was implemented in MrBayes 3.2.6 [40]. Sequences *ITS*, *LSU* rDNA, *coxI*, and *coxII* were defined as four independent partitions, each estimated using its own model parameters. The best-selected model was applied to each partition. The Markov Chain Monte Carlo (MCMC) chain was run for 2,000,000 generations, with the STOPRULE command set to STOPVAL = 0.01 and trees sampled every 100 generations. Chain convergence was verified using Tracer 1.5 (http://tree.bio.ed.ac.uk/software/tracer accessed on 12 February 2026) to ensure sufficiently large effective sample size (ESS) values > 200. The combined tree was summarized using the sump and sumt commands with a 25% burn-in.

Phylogenetic trees were rooted at the midpoint, implemented as the default rooting option in both MrBayes and RAxML analyses. Given that the genus *Phytopythium* is phylogenetically intermediate between *Phytophthora* and *Pythium* [29], no single outgroup was specified; instead, midpoint rooting was applied to all inferred trees.

All sequences obtained in this study have been deposited in GenBank, and the relevant information is provided in Table 2 (*ITS*: PZ022717.1 and PZ022718.1; *LSU*: PZ022833.1 and PZ022834.1; *coxI*: PZ284476 and PZ284477; *coxII*: PZ284474 and PZ284475).

### 2.4. Pathogenicity Testing of the Pathogen

Pathogenicity tests were conducted using the two representative isolates HNJC71 and HNJC90 on rubber tree seedlings cultivated in a greenhouse and bag-grown seedlings indoors. Healthy tender branches of rubber trees in the greenhouse were slightly wounded on the surface using a sterilized toothpick. The wounds were then inoculated with mycelium of the test strain. Healthy branches inoculated with sterile PDA blocks served as negative controls. The inoculated sites were first wrapped with sterile, moistened cotton balls and then sealed tightly with plastic wrap to maintain humidity. Each treatment was replicated three times. For indoor bag-grown seedlings, artificial inoculation was performed by spraying a spore suspension containing approximately 10^5^ sporangia/mL. To prepare the sporangial suspension, rubber tree leaf pieces (5 cm × 5 cm) were surface-sterilized with 70% ethanol for 5 min, rinsed three times with sterile distilled water, and placed onto PDA plates for subsequent inoculation with the tested strains [41,42]. The tested strains were then inoculated onto the leaf pieces and incubated at 25 °C in the dark for 6 days to allow mycelial colonization and abundant sporangium production. Sporangia were harvested by washing the colonized leaf surfaces with sterile distilled water. The resulting suspension was filtered through a nylon filter sieve (pore size: 25–30 μm) to remove mycelial debris. Sporangia were counted using a hemocytometer, and the concentration was adjusted to approximately 10^5^ sporangia/mL with sterile distilled water. Plants sprayed with sterile water served as negative controls. After inoculation, the bag-grown seedlings were covered with plastic bags to maintain humidity. The two inoculation methods were selected for complementary purposes. The toothpick wound and mycelial plug method was employed to fulfill Koch’s postulates by ensuring direct contact between the pathogen and host tissues, allowing reliable re-isolation from symptomatic tissues. The spore suspension spray method was used to simulate natural infection conditions, as *P. vexans* is naturally dispersed via zoospores in water (rain splash or irrigation), providing additional evidence that the pathogen can infect without artificial wounding. The branches were observed regularly for symptom development, and the results were documented by photography.

After the artificially inoculated plants developed disease symptoms, pathogen re-isolation was performed following Koch’s postulates. Diseased tissue blocks of approximately 0.5 cm × 0.5 cm were excised from the interface between diseased and healthy tissue at the inoculation site. After surface disinfection with 75% ethanol, the tissue blocks were transferred onto selective PDA medium. The consistency of the re-isolated strains was confirmed through morphological and molecular identification. The primers used for molecular identification included those targeting the *ITS*, *LSU* rDNA, *coxI*, and *coxII*, as listed in Table 1.

### 2.5. Fungicide Sensitivity Testing of the Pathogen

The toxicities of six fungicides were evaluated using a mycelial growth inhibition assay against the two representative isolates HNJC71 and HNJC90. PDA (20 mL) was amended with fungicides dissolved in 0.5% (*v*/*v*) dimethyl sulfoxide (DMSO) was used to achieve the following final concentrations: 96% metalaxyl or metalaxyl·propamocarb at 0.04, 0.08, 0.16, 0.32, 0.64, 1.28, and 2.56 μg/mL; dimethomorph at 4.0, 8.0, 16.0, 32.0, 64.0, 128.0, and 256.0 μg/mL; the 96% pyrimethanil or flupyrimethanil at 2.0, 4.0, 8.0, 16.0, 32.0, 64.0, and 128.0 μg/mL; and 91.7% fenoconazole at 5.0, 10.0, 20.0, 40.0, 80.0, 160.0, and 320.0 μg/mL. Control plates contained 0.5% DMSO only. The DMSO concentration was maintained at 0.5% (*v*/*v*) in all fungicide-amended media and in the control plates. Mycelial plugs (5 mm in diameter) were cut from the margins of 3-day-old colonies of strains 90 and 71 and placed in the center of each PDA plate. The plates were sealed with parafilm and incubated in darkness at 24 ± 1 °C. Three replicate plates were used for each concentration. After 3 days of incubation, radial mycelial growth was measured. The half-maximal effective concentration (EC_50_) and the relative growth inhibition rate (%) were calculated using the formula provided below. EC_50_ values were calculated by linear regression of probit-transformed percentage inhibition values against log_10_-transformed fungicide concentrations, following the method described by Finney (1971) [43]. The regression equations in the form *y* = a*x* + b, where *y* represents the probit-transformed inhibition rate and *x* represents the log_10_-transformed concentration.

(1) $$mycelial\;growth\;inhibition\;(\%)=\frac{C-T}{C}\times 100$$

where C and T are the average diameter (mm) of fungal mycelia in the control and treatment, respectively.

### 2.6. Data Processing

All experiments were performed with three independent biological replicates, each with three technical replicates. Data are expressed as mean ± standard deviation (SD). Significant differences (*p* < 0.05) were determined using one-way analysis of variance (ANOVA) followed by Duncan’s new multiple range test in SPSS Statistics 20.

## 3. Results

### 3.1. Field Disease Symptoms and Oomycete Isolation and Purification

**Figure 1:**
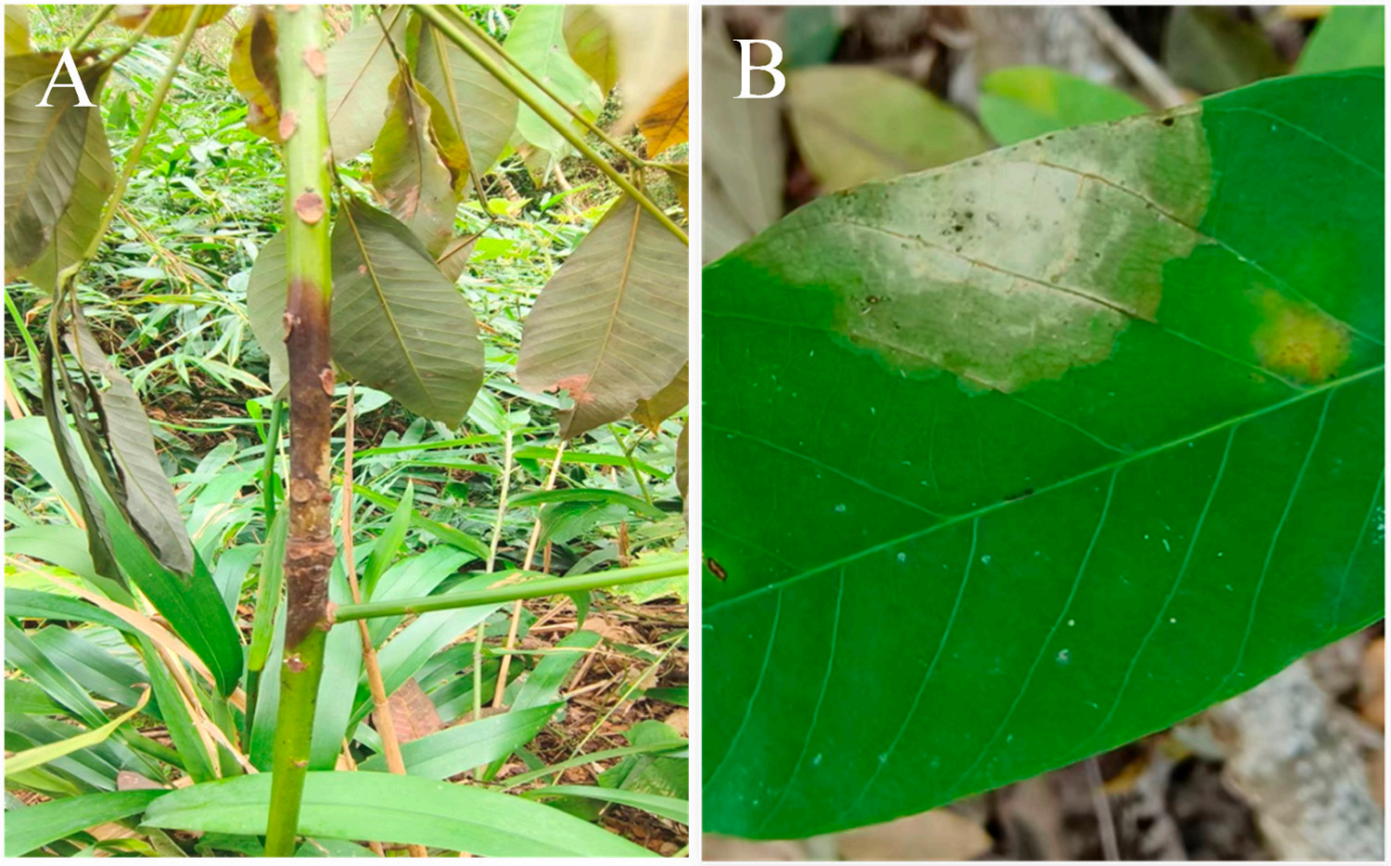
Field symptoms of diseased rubber trees. (**A**) Brown necrotic lesions on green, non-lignified stems of rubber seedlings. (**B**) Brown, water-soaked necrotic lesions on rubber tree leaves.

Affected tender branches and green stems of rubber tree seedlings developed black, water-soaked necrotic lesions. Under conditions of high temperature and high humidity, these lesions continuously expanded outward, eventually forming a black, annular necrosis encircling the stem, which disrupted nutrient transport. Internally, the xylem appeared blackened. Leaves on affected branches wilted, drooped, and eventually abscised. Young leaves, after infection, initially exhibited dark green, water-soaked lesions, sometimes accompanied by white gel exudation from the affected areas. Subsequently, the infected leaves turned yellow, wilted, and abscised (Figure 1).

From these samples, five oomycete strains with colony characteristics resembling *Phytophthora* but exhibiting rapid growth were isolated. Strains HNJC71 and HNJC90 were selected for further study. The remaining three strains (HNJC73, HNJC87, and HNJC89) were confirmed as *P. vexans* by *ITS* sequencing but were not subjected to detailed morphological and physiological characterization.

### 3.2. Molecular Identification and Phylogenetic Analysis

All five isolates were confirmed as *P. vexans* by *ITS* sequencing. For the two representative isolates HNJC71 and HNJC90, full four-locus sequences (*ITS*, *LSU* rDNA, *coxI*, and *coxII*) were obtained and used for the multi-gene phylogenetic analysis. Through PCR amplification and bidirectional sequencing, the *ITS*, *LSU* rDNA, *coxI*, and *coxII* gene fragments of the tested strains HNJC71 and HNJC90 were obtained, with lengths of 789 bp, 707 bp, 627 bp, and 563 bp, respectively. All sequences obtained from strains HNJC71 and HNJC90 in this study have been submitted to the GenBank database. BLAST v2.11.0 analysis was performed against publicly available *Phytopythium* sequences in GenBank to search for closely related species with high homology. The accession numbers of strains HNJC71 and HNJC90 are listed in Table 2.

For phylogenetic tree construction, a total of 294 sequences from 36 strains were used (Table 2). The phylogenetic analyses were based on sequences from four genomic regions: the *ITS*, *LSU* rDNA, *coxI*, and *coxII*.

Two different phylogenetic methods were employed to align and analyze the individual gene sequences, after which the four-gene dataset was used to construct the phylogenetic tree. Since comparison of the individual gene trees revealed no significant topological conflicts, the *ITS*, *LSU* rDNA, *coxI*, and *coxII* sequence datasets were combined and concatenated. The cluster analysis used sequences downloaded from the National Center for Biotechnology Information (NCBI) database. The concatenated matrix comprised 2686 bp of nucleotides.

**Figure 2:**
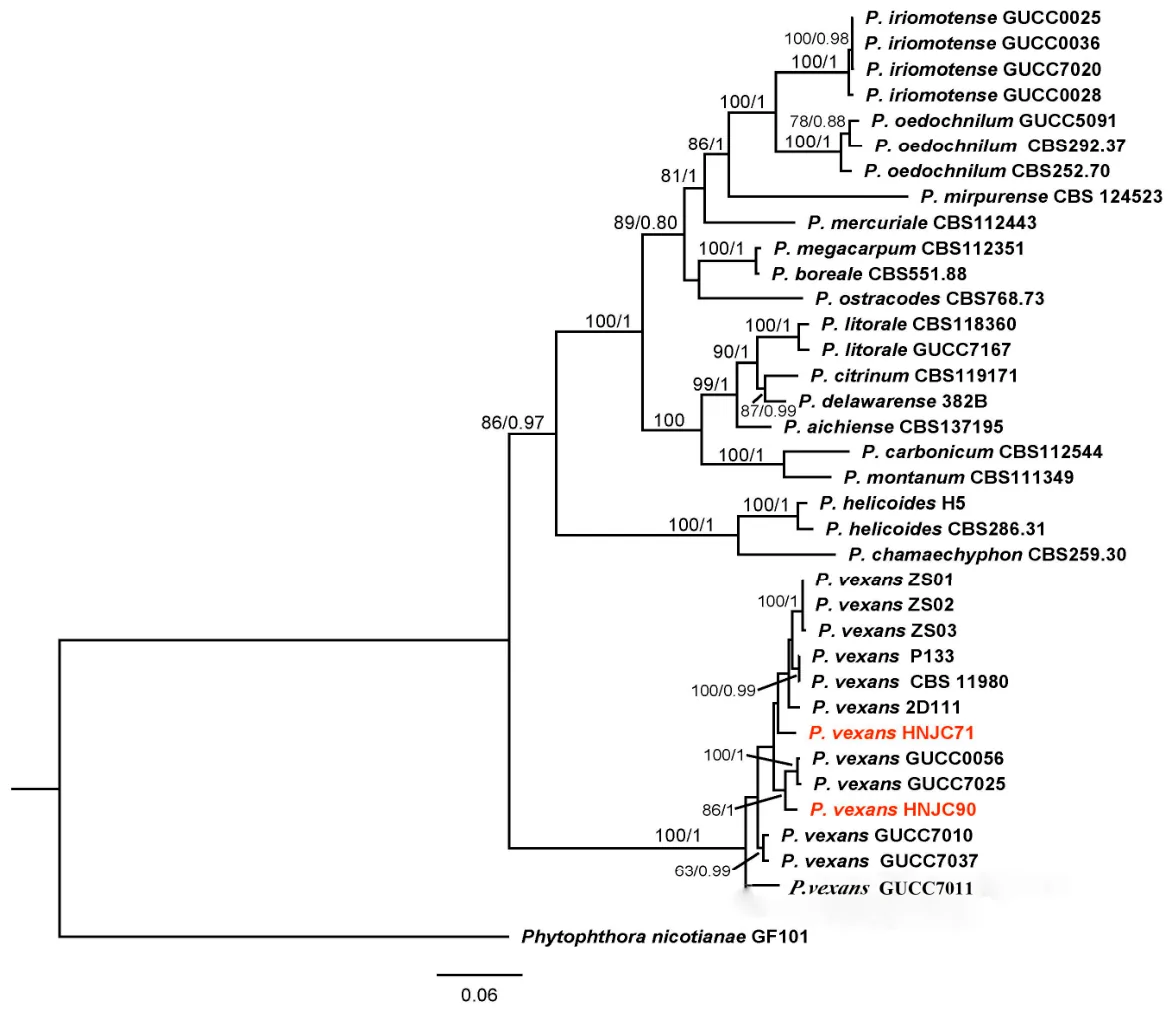
A phylogenetic tree was constructed based on BI of the concatenated *ITS*, *LSU* rDNA, *coxI*, and *coxII* sequence dataset, representing all currently accepted *Phytopythium* species. Node support values are indicated as ML bootstrap support (MLBS)/Bayesian posterior probability (BPP). Isolates obtained in this study are highlighted in red font, including HNJC71 and HNJC90, which were used in the fungicide sensitivity assays. Support values (MLBS < 70% or BPP < 0.70) are not displayed.

The phylogenetic tree topologies obtained from BI and ML analyses were largely congruent, indicating that the evolutionary relationships of the isolated strains were statistically supported (Figure 2). The phylogenetic analysis revealed that the strains isolated from rubber tree samples clustered with the reference strain of *P. vexans* with strong statistical support. Specifically, strains HNJC71 and HNJC90 were closely related to *P. vexans* and formed a clade with *P. vexans* strains, with a bootstrap value (BP) of 100% and a Bayesian posterior probability (BPP) of 1. All isolates obtained in this study showed high similarity to previously reported *P. vexans* strains. Therefore, the pathogen was conclusively identified as *P. vexans*.

### 3.3. Morphological Identification and Biological Characteristics

**Figure 3:**
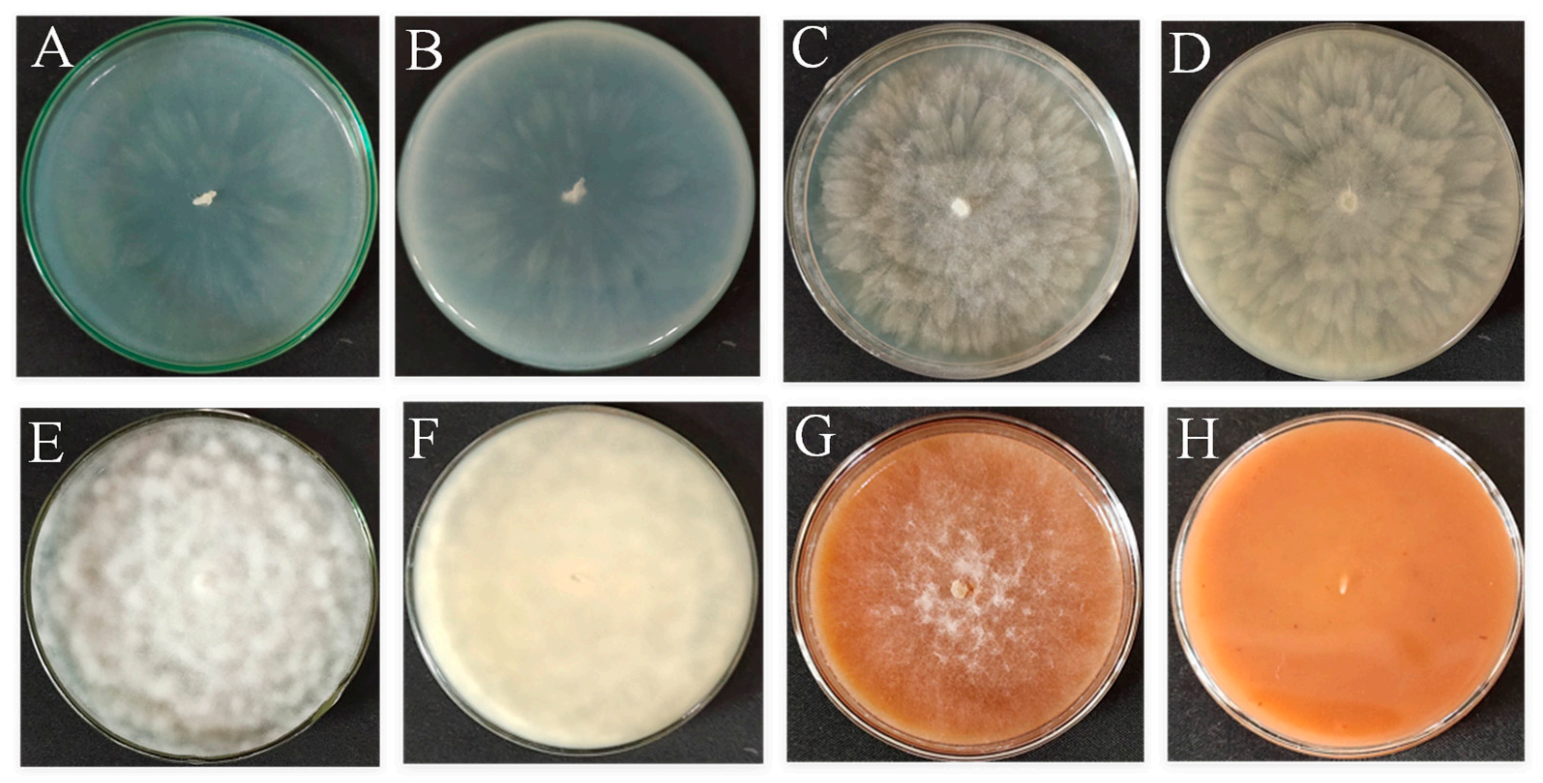
Colony formation of strain HNJC90 isolated from rubber tree cultured after 3 days on different media at 25 °C. (**A**) Top and (**B**) reverse views of colony morphology on MEA medium. (**C**) Top and (**D**) reverse views of colony morphology on CA medium. (**E**) Top and (**F**) reverse views of colony morphology on PDA medium. (**G**) Top and (**H**) reverse views of colony morphology on V8-agar medium.

Strains HNJC71 and HNJC90 grew rapidly on CA and PDA. On MEA, both strains formed white colonies with sparse aerial mycelia and no obvious radial pattern (Figure 3A,B). On CA, the colonies appeared white, with abundant aerial mycelia exhibiting a chrysanthemum-like radial growth pattern (Figure 3C,D). The best growth was observed on PDA, where the colonies displayed fluffy, dense mycelia with a lamb’s wool-like appearance (Figure 3E,F). On V8 agar, the isolates formed white colonies with sparse aerial mycelia and no distinct pattern (Figure 3G,H). Notably, the abundance of aerial mycelia varied depending on the culture medium: mycelia were abundant and exhibited chrysanthemum-like radial growth on CA, most dense and abundant on PDA, less abundant on V8 agar, and least abundant on MEA.

**Figure 4:**
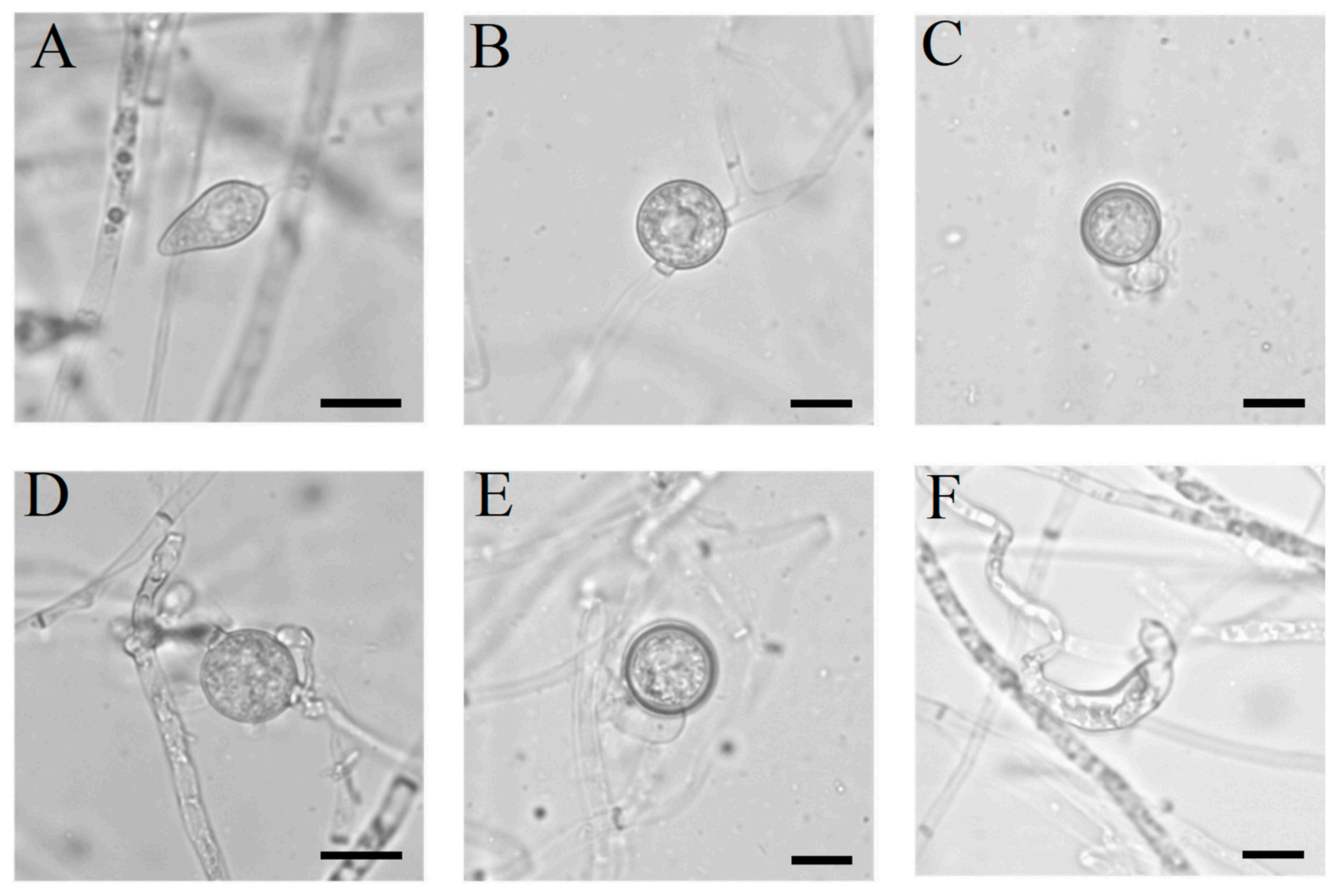
Morphological characteristics of *Phytopythium vexans*. (**A**) Sporangia showing distinct papillate structures. (**B**,**C**) Oospores with smooth walls and abundant matrix material. (**D**,**E**) Antheridium in contact with oogonium. (**F**) Antheridium. Scale bars: 10 μm (applies to all panels).

The sporangia of these isolates were spherical to oblong-ovoid in shape, with smooth surfaces, abundant internal matrix material, and distinct papillate structures (or protrusions) (Figure 4A). Empty sporangia were also observed (Figure 4A). For strain HNJC90, the average sporangium size was 13.48 μm × 11.57 μm, with dimensions ranging from 12.91 μm to 17.18 μm × 8.09 μm –17.23 μm; for strain HNJC71, the average sporangium size was 13.19 μm × 12.41 μm, with dimensions ranging from 12.62 μm to 16.36 μm × 8.57 μm –16.45 μm (*n* = 100). Some oospores were surrounded by oogonia or exhibited short protrusions. Oogonia were spherical, smooth-walled, and terminal, occasionally intercalary (Figure 4B–E). Oogonial diameter ranged from 14.23 μm to 27.79 μm (mean = 21.52 μm) for strain HNJC90, and from 14.88 μm to 28.12 μm (mean = 22.34 μm) for strain HNJC71. Antheridia were irregular in shape, terminal, predominantly monoclinous, and rarely diclinous (Figure 4F). A summary of morphometric data for both isolates is presented in Table 3. Based on colony morphology, color, and sporangial characteristics, these isolates were identified as *P. vexans*.

**Figure 5:**
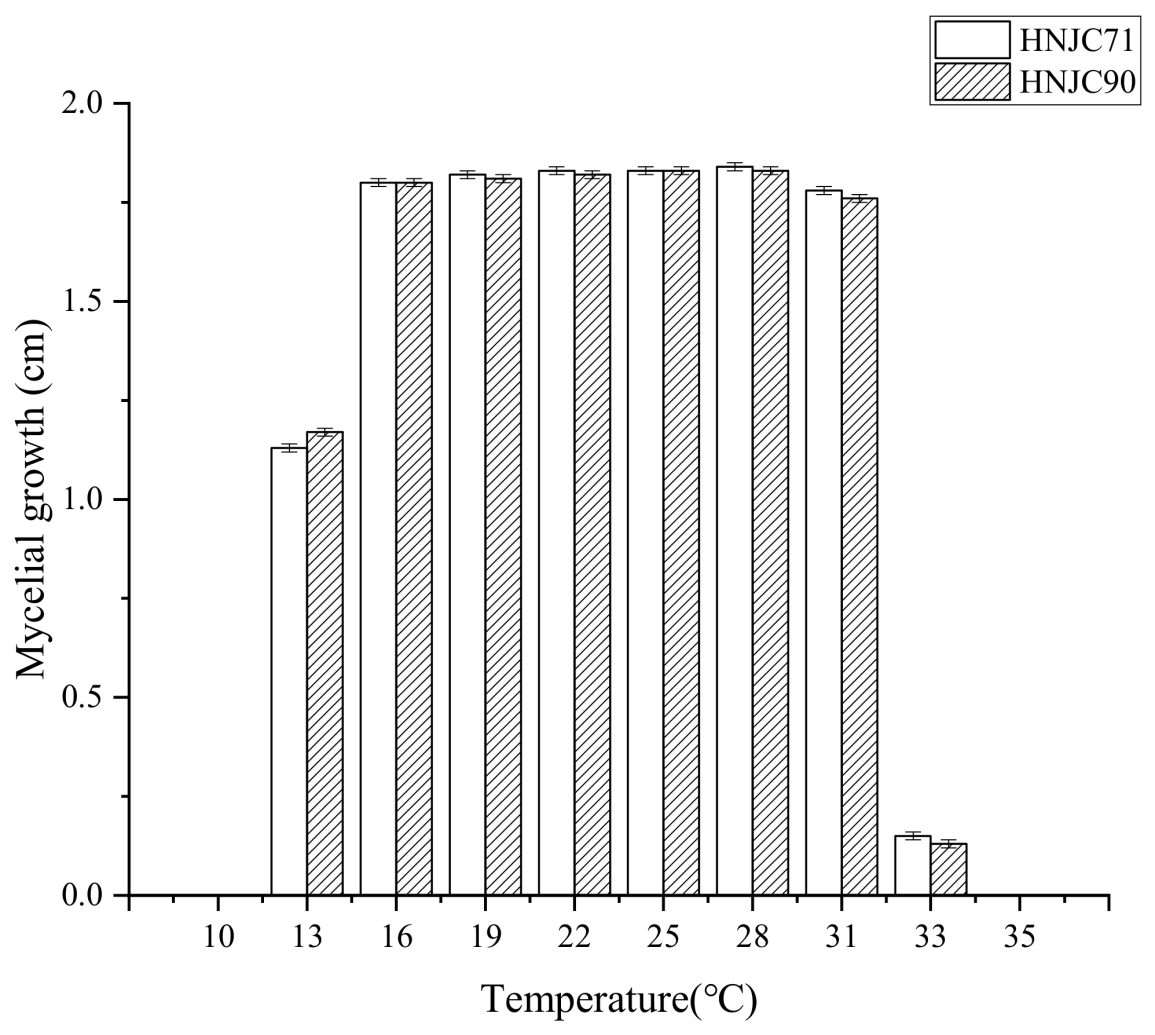
Effect of temperature on colony diameter growth of isolates HNJC71 and HNJC90 after 3 days of culture on PDA medium. Data are shown as mean ± SD (*n* = 3). Different lowercase letters above the data points indicate significant differences among temperatures for each strain (one-way ANOVA, Duncan’s multiple range test, *p* < 0.05). No significant difference was observed between the two strains at any temperature (*p* > 0.05).

Strains HNJC71 and HNJC90 were capable of growing at temperatures ranging from 13 °C to 33 °C, with the optimal growth temperature range being 16–31 °C (Figure 5). At 28 °C, both strains reached maximum colony diameters (HNJC71: 78.5 ± 2.3 mm; HNJC90: 76.8 ± 2.8 mm), and no significant difference was observed between the two strains at any temperature tested (*p* > 0.05). One-way ANOVA revealed that temperature had a significant effect on mycelial growth for both strains (*p* < 0.001). Within the range of 16–31 °C, the mycelia of the colonies were most dense and fluffy. At 33 °C, mycelial growth was inhibited, and at 35 °C, no growth occurred (Figure 5).

### 3.4. Pathogenicity Testing

**Figure 6:**
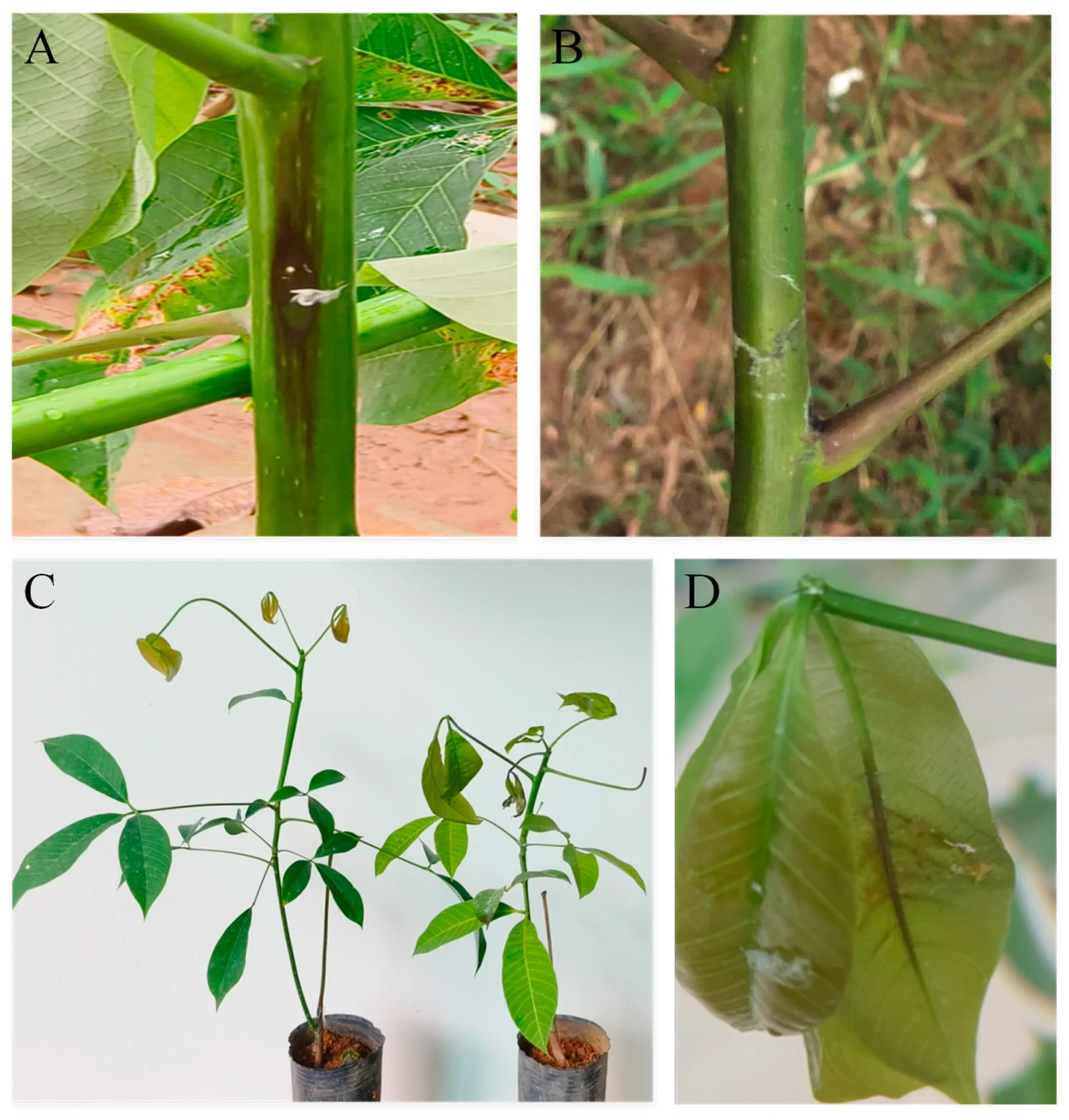
Symptoms of brown stripe on stems and brown, water-soaked necrosis on leaves of rubber seedlings after inoculation. (**A**) Diseased rubber seedling showing brown stripe symptoms on the stem at 21 days post-inoculation (dpi) in a greenhouse in Jinghong, Xishuangbanna. (**B**) Control rubber seedling without brown stripe symptoms on the stem at 21 dpi in the greenhouse. (**C**) Control seedling with green leaf (left) and diseased seedling with leaf chlorosis (right) in the laboratory at 12 dpi. (**D**) Water-soaked necrosis on leaves of rubber seedlings in the laboratory at 7 dpi. dpi: days post-inoculation.

Seven days after inoculation of greenhouse-grown rubber tree seedlings with strains HNJC71 and HNJC90, light blackish-brown, elongated lesions appeared on the stems (Figure 6A). In contrast, no symptoms were observed in the control group. At 14 days post-inoculation, the control plants grew vigorously, with bright green leaves spreading outward (Figure 6B). In contrast, the inoculated plants exhibited leaf drooping, yellowing, and wilting symptoms. Thereafter, at 21 days post-inoculation, the symptoms progressively intensified, with the degree of wilting increasing daily.

In the greenhouse inoculation experiments using wound inoculation with mycelial plugs, all three replicate branches per strain developed symptoms at 7 days post-inoculation (dpi), indicating a latent period of 7 days, with disease incidence reaching 100% for both strains. At 7 dpi, the mean lesion length was 1.54 mm for strain HNJC71 and 1.96 mm for strain HNJC90. At 14 dpi, lesions expanded progressively, with mean lesion lengths of 4.68 mm (HNJC71) and 4.92 mm (HNJC90), and leaves began to droop and show chlorosis. At 21 dpi, lesions encircled the inoculation site in all replicates, with wilting and drooping of leaves above the inoculation point; mean lesion lengths reached 9.35 mm (HNJC71) and 10.18 mm (HNJC90). Disease severity, assessed as the percentage of leaf area showing chlorosis and necrosis, ranged from 60% to 85% at 21 dpi across all replicates.

The results of the inoculation tests using bag-grown seedlings were similar to those obtained in the greenhouse. Compared with the control bag-grown seedlings, the inoculated rubber tree seedlings showed leaf yellowing, while the uninoculated rubber seedlings grew healthily and remained asymptomatic (Figure 6C). The inoculated seedlings developed brown, water-soaked lesions on the leaves (Figure 6D). For the indoor bag-grown seedling experiments using spore suspension spray inoculation, no symptoms were observed at 2 dpi (0% incidence). At 4 dpi, sporadic mild symptoms appeared, with disease incidence reaching 15.2% for strain HNJC71 and 17.6% for strain HNJC90. At 7 dpi, the number of symptomatic plants increased significantly, with some leaves showing water-soaked lesions; disease incidence rose to 73.2% (HNJC71) and 76.7% (HNJC90). At 9 dpi, disease development stabilized, and final disease incidence reached 100% for both strains, with typical water-soaked necrotic lesions on leaves (Figure 6D). No symptoms were observed in the control group throughout the experiment (0% incidence), and the uninoculated seedlings grew healthily (Figure 6C). The cultural and morphological characteristics of the re-isolated strains from symptomatic tissues were identical to those of the original isolates. The DNA sequences of the strains re-isolated from the inoculated rubber tree tissues were consistent with those of the isolates used for inoculation. The symptoms observed on the artificially inoculated rubber trees were similar to those observed in the field, thereby fulfilling Koch’s postulates. A summary of the quantitative pathogenicity data is presented in Table 4.

### 3.5. Susceptibility of Phytopythium Isolates to Fungicides

**Figure 7:**
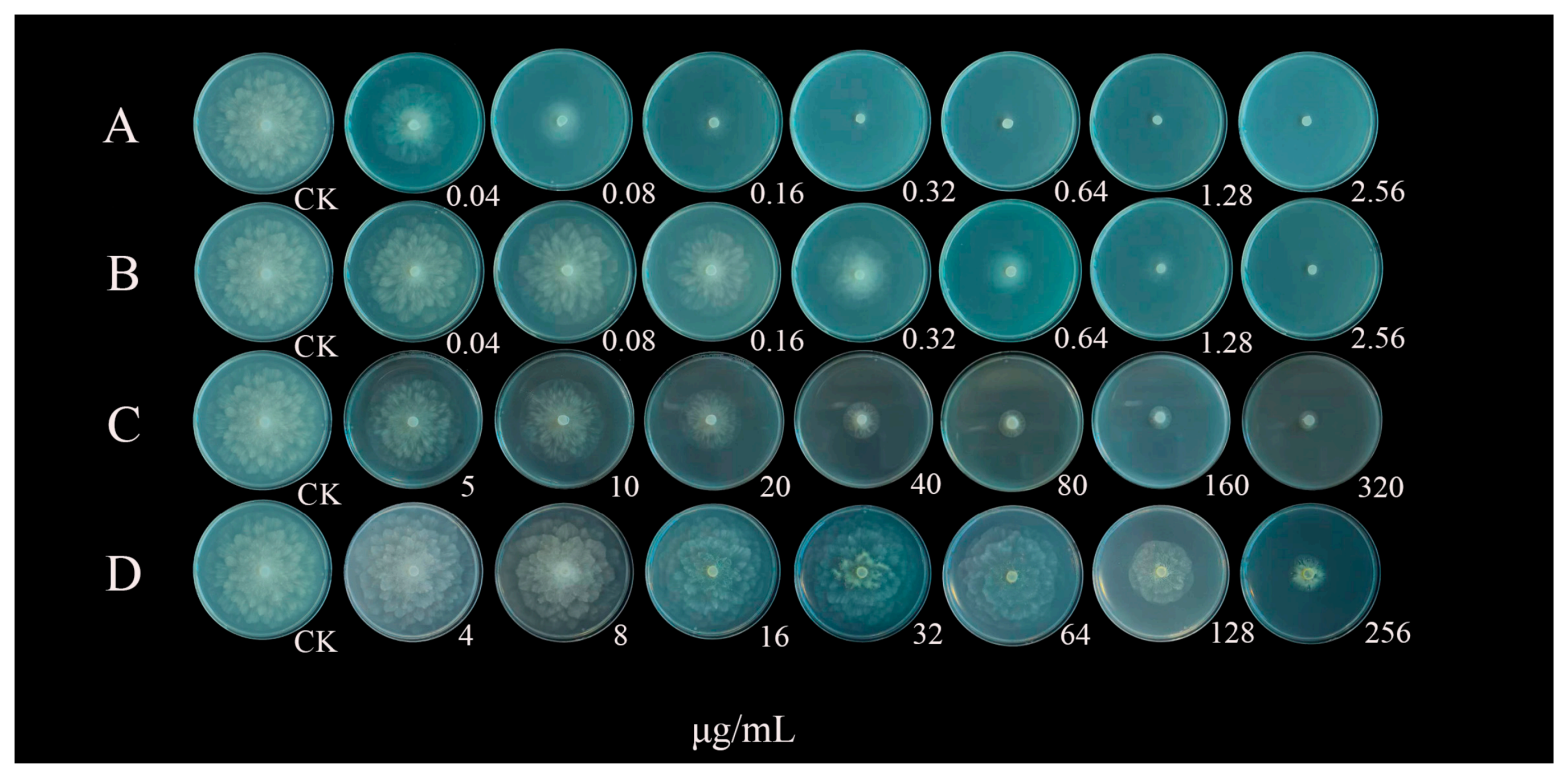
Inhibitory effects of six different fungicides at corresponding doses on the growth of isolate HNJC71 on fresh PDA medium after 3 days of culture. Data are expressed as mean ± SD (*n* = 3). (**A**) 96% Metalaxyl. (**B**) Metalaxyl·propamocarb. (**C**) 91.7% Fenoconazole. (**D**) Dimethomorph.

Regarding chemical control, the 96% metalaxyl demonstrated exceptional inhibitory activity, yielding EC_50_ values as low as 0.04 µg/mL (95% confidence interval: 0.03–0.05) for strain HNJC90 and 0.06 μg/mL (0.05–0.07) for strain HNJC71 (Table 5). The metalaxyl·propamocarb mixture also demonstrated strong inhibitory activity, with EC_50_ values of 0.43 μg/mL (0.40–0.46) for HNJC71 and 0.44 μg/mL (0.41–0.47) for HNJC90. The 91.7% fenoconazole showed moderate and comparable activity against both isolates, with EC_50_ values of 18.64 μg/mL (16.29–21.12) and 19.50 μg/mL (17.12–22.03), respectively. Dimethomorph exhibited relatively low inhibitory activity, with EC_50_ values of 134.16 μg/mL (124.34–145.50) for HNJC90 and 140.60 μg/mL (124.06–161.84) for HNJC71 (Figure 7). All dose–response models showed a good fit (***r^2^*** values ranging from 0.91 to 0.99) (Table 5).

## 4. Discussion

The central novelty of this study lies in the first report of *Phytopythium vexans* causing stem and leaf necrosis on rubber trees. A previously undocumented disease manifestation on above-ground young organs (tender stems and young leaves) that expands the known pathogenic niche of this oomycete on *H. brasiliensis* and challenges the traditional paradigm that *Phytophthora* species are the sole oomycete pathogens of aerial tissues of rubber trees. Through field investigation, pathogen isolation, molecular and morphological identification, pathogenicity testing, and fungicide sensitivity assays, *P. vexans* was confirmed as the causal agent of blackish-brown necrosis on tender branches and leaves. The three hypotheses proposed in the introduction were systematically validated: multi-gene phylogeny unequivocally placed the isolates within *P. vexans* (Hypothesis i); Koch’s postulates confirmed their pathogenicity on rubber seedlings (Hypothesis ii); and fungicide sensitivity assays supported the prediction that metalaxyl exhibits the strongest inhibitory activity (Hypothesis iii).

It should be noted that *Pythium vexans* (currently reclassified as *Phytopythium vexans*) was previously reported on rubber trees in Hainan, China, causing patch canker on trunks and roots, with subspherical, non-papillate sporangia [44]. In contrast, the isolates in this study produced distinctly papillate sporangia and infected aerial tissues—specifically tender, non-lignified stems and young leaves—causing necrotic lesions, defoliation, and branch dieback. This represents a distinct disease manifestation on above-ground young organs, which has not been previously documented for *P. vexans* on rubber trees. The observed differences in sporangial morphology, infected tissue types, and disease outcomes suggest that *P. vexans* may exhibit pathogenic plasticity on *H. brasiliensis*, warranting further investigation into the potential existence of host- or tissue-specialized lineages.

The multi-gene phylogenetic analysis (*ITS*, *LSU*, *coxI*, and *coxII*) provided robust support for species identification (BP = 100%, BPP = 1.0). While *P. mirpurense* shares high *ITS* and *LSU* similarity with *P. vexans* within clade K [29,45], and *P. vexans* isolates are known to be heterogeneous in *ITS* sequences [46], the concatenated four-gene approach—combining nuclear and mitochondrial markers—substantially increased phylogenetic resolution compared with single-locus analyses [29,45]. The combined *coxI* and *coxII* phylogenies, in particular, have been shown to reveal subclades not resolved by *ITS* alone [29]. Thus, the concatenated analysis strongly supports identifying our isolates as *P. vexans*.

Morphological and biological characterization further supported the molecular identification. Colony morphology and sporangial characteristics were consistent with typical *P. vexans* features [47,48]. Temperature adaptability assays showed growth at 15–30 °C, with an optimum of 25–30 °C [12], consistent with the climatic conditions of tropical rubber-growing regions [25]. ISSR-based population studies have revealed significant genetic diversity among *P. vexans* isolates from different continents, with low gene flow between geographically distinct populations, suggesting that *P. vexans* may represent a species complex [49]. In China, *P. vexans* has been increasingly reported on diverse hosts, including ramie, camphor tree, macadamia, durian, apple, and *Rhododendron simsii* [12,47,48,50,51,52], indicating that the pathogen is well-established in Chinese agro-ecosystems. Whether Chinese isolates represent indigenous or introduced populations remains unclear, highlighting the need for population genetic studies to trace dissemination pathways.

Pathogenicity tests confirmed that *P. vexans* isolates HNJC71 and HNJC90 induced typical symptoms on rubber seedlings, fulfilling Koch’s postulates. Notably, *P. vexans* mainly affected tender branches and seedlings, consistent with previous observations that it more readily infects young host tissues [53]. However, because the experimental setup did not include comparative inoculations of mature tissues, this finding remains an observational tissue preference rather than a quantitatively tested conclusion. The specific virulence factors involved in the rubber tree-*P. vexans* interaction remain unknown and require future investigation through histological and transcriptomic analyses.

Fungicide sensitivity assays revealed that 96% metalaxyl exhibited the strongest inhibitory activity (EC_50_ = 0.04–0.06 μg/mL), followed by the metalaxyl·propamocarb mixture. This is consistent with the recognized efficacy of metalaxyl as a systemic oomycete-specific fungicide [54]. The metalaxyl-propamocarb mixture also exhibited high inhibitory activity, supporting the strategy of using fungicide mixtures to improve efficacy and delay resistance [55]. In contrast, dimethomorph exhibited relatively weak inhibition (EC_50_ = 134–141 μg/mL), consistent with reports of dimethomorph insensitivity in *P. vexans* [56]. The EC_50_ values obtained in this study are comparable to those reported for other *P. vexans* isolates, such as from camphor tree (most sensitive to metalaxyl hymexazol) and soybean-associated *Phytopythium* species (EC_50_ < 10 μg/mL for metalaxyl) [12,57]. Our isolates (EC_50_ = 0.04–0.06 μg/mL) fall well within the sensitive range reported for other *Phytopythium* species, indicating that the two isolates tested are highly sensitive to metalaxyl in vitro. However, conclusions regarding the resistance status of the broader *P. vexans* population on rubber trees would require analysis of a substantially larger collection of isolates.

While metalaxyl showed strong in vitro activity, it should not be perceived as a standalone definitive control method. Metalaxyl is a single-site mode of action fungicide (FRAC Group 4), and resistance selection pressure is well documented across oomycete species, including *P. vexans* [46,58]. Therefore, the EC_50_ values reported here should be interpreted as baseline sensitivity data for initial screening rather than as direct field recommendations. For sustainable management, the following strategies are recommended: rotate metalaxyl with fungicides of different modes of action, use mixture formulations (e.g., metalaxyl-propamocarb), apply fungicides at the recommended rates to avoid sublethal doses, limit the number of consecutive applications of fungicides from the same FRAC group, and integrate chemical control with cultural practices. Future field trials should evaluate both efficacy and resistance risk under natural infection conditions.

It is important to emphasize that the EC_50_ values were obtained under standardized in vitro conditions and should not be directly extrapolated to predict field efficacy. Factors such as environmental variables, host physiology, fungicide formulation, and application methods may influence field performance. Furthermore, EC_50_ values based on mycelial growth inhibition do not capture the full spectrum of fungicide activity against sporangia and zoospores—the primary infectious propagules under natural conditions [12,46]. Future investigations should include sporangial germination assays and field trials to validate practical efficacy. The use of DMSO (0.5% *v*/*v*) as a solvent was applied uniformly across all treatments, minimizing solvent-induced bias, though its potential contribution to observed effects cannot be completely excluded.

The broader significance of these findings extends beyond adding a new host record. This discovery highlights the vulnerability of rubber plantations to previously overlooked oomycete pathogens, provides essential molecular reference markers for rapid detection, and offers actionable guidance for fungicide selection. The identification of *P. vexans* on rubber trees raises important questions for future research, including potential interactions with *Phytophthora* species in disease complexes, and the possible role of climate change or agronomic practices in driving host shifts. Addressing these questions will require broader geographic surveys, longitudinal field observations, and comparative genomics.

Understanding the potential spread of *P. vexans* between rubber plantations is critical for epidemiological risk assessment. The pathogen produces sexual oospores for long-term survival and motile zoospores that readily disperse through water, facilitating rapid local spread [45,46]. The humid tropical climate of Yunnan and Hainan, with prolonged monsoon rainfall and high humidity, promotes zoospore release and host colonization [46]. Anthropogenic activities—including movement of contaminated soil via machinery, tools, footwear, and nursery stock—play a significant role in inter-plantation spread [56]. Given its broad host range across multiple continents [41,46], *P. vexans* may persist in alternative weed hosts or nearby crops, serving as a continuous reservoir for reinfection.

Nurseries and young plantations are particularly vulnerable to *P. vexans* infection. Based on the biology of oomycetes and general disease management principles reported in the literature [41,46,56], the following preventive measures are recommended: (i) regular monitoring during the rainy season; (ii) cultural practices such as improving drainage, avoiding excessive planting density, pruning to reduce humidity, and removing infected debris; (iii) using drip irrigation and disinfecting tools regularly with 70% ethanol; (iv) evaluation of metalaxyl-based products in future field trials to determine their efficacy and optimal application timing under natural infection conditions, with consideration of rotation with fungicides of different modes of action; and (v) using certified disease-free planting materials and quarantining new introductions. The effectiveness of these measures specifically against *P. vexans* in rubber plantations requires further field validation.

Several limitations of this study should be acknowledged. Only five isolates were obtained, and detailed characterization was performed on two representative strains (HNJC71 and HNJC90). While sufficient for a first report, the limited sample size precludes a comprehensive understanding of the genetic and phenotypic diversity within the *P. vexans* population affecting rubber trees. In particular, the fungicide sensitivity data were obtained from only two isolates; given the intraspecific variability discussed above [46,49], these results should not be generalized to the broader *P. vexans* population without further validation using a larger collection of isolates. Additionally, sampling from two provinces (Yunnan and Hainan) was not spatially extensive enough to assess population structure or geographic variation in virulence and fungicide sensitivity. Future studies should include larger collections from multiple locations across different rubber-growing regions to evaluate spatial variability, population genetics, and the potential existence of host-specialized lineages. Long-term field surveys are also needed to monitor disease dynamics and assess the impact of environmental factors on disease development.

## 5. Conclusions

In this study, we identified *P. vexans* as a novel pathogen causing blackish-brown necrosis on tender stems and leaves of rubber trees (*H. brasiliensis*) through field symptom investigation, pathogen isolation, morphological observation, molecular phylogenetic analysis (based on *ITS*, *LSU* rDNA, *coxI*, and *coxII* sequences), and pathogenicity tests fulfilling Koch’s postulates. The pathogen exhibited optimal mycelial growth at 16–31 °C, with no growth at 35 °C, indicating its adaptation to tropical and subtropical rubber-growing regions. Fungicide sensitivity assays revealed that 96% metalaxyl showed the strongest inhibitory activity (EC_50_ values as low as 0.04 μg/mL and 0.06 μg/mL), followed by the metalaxyl·propamocarb mixture, whereas dimethomorph exhibited the weakest activity. Although these in vitro results are promising, field trials are required to validate the efficacy of these fungicides under natural infection conditions before recommending their widespread use in plantations. This study provides the first report of *P. vexans* causing stem and leaf necrosis on rubber trees, enriching the understanding of oomycete diseases affecting aerial tissues of *H. brasiliensis* and providing a theoretical basis for accurate disease diagnosis, epidemiological monitoring, and the development of effective chemical control strategies. Our findings also highlight the need for continuous surveillance of *P. vexans* in rubber plantations to mitigate its potential threat to natural rubber production.

## Figures and Tables

**Table 1:** Primer sequences used for molecular identification of isolates.

Primer	Sequence (5′-3′)	Annealing Temp.
ITS1	TCCGTAGGTGAACCTGCGG	55
ITS4	TCCTCCGCTTATTGATATGC	55
Coxlevup	TCAWCWMGATGGCTTTTTCAATTC	53
Coxlevlo	CYTCHGGRGTWGWCAAAAAACCAAA	53
CoxII-F	GGCAAATGGGGTTTTCAACAGATC	55
CoxII-Rc4	TGATTWAYNCCACAAATTICTCATATTG	55
NL1	GCATATCAATAAGCGGAGGAAAAG	53
NL4	GGTCCGTGTTTCAAGACGG	53

**Table 2:** GenBank accession numbers of isolates used for phylogenetic analysis in this study.

Species	Isolates	GenBank Accession Numbers			
		rDNA *ITS*	*LSU* rDNA	*coxI*	*coxII*
*P. aichiense*	CBS 137195	AB948197	AB948194	AB948191	AB948192
*P. chamaechyphon*	CBS 25930	AB690609	AB690593	AB690644	AB690674
*P. carbonicum*	CBS 112544	AB725876	AB996605	AB690648	AB690678
*P. citrinum*	CBS 119171	AY197328	AB690597	AB690649	AB690679
*P. delawarense*	382B	AB725875	AB690591	AB690642	AB690672
*P. helicoides*	CBS 28631	AB725878	AB690594	AB690645	AB690675
	H5	AB690611	AB690582	AB690633	AB690663
*P. iriomotense*	GUCC0025	AB690622	AB690600	AB690652	AB690682
	GUCC0028	AB690623	AB690601	AB690653	AB690683
	GUCC0036	AB690624	AB690602	AB690654	AB690684
	GUCC7020	AB690629	AB690607	AB690659	AB690689
*P. litorale*	GUCC7167	AB690612	AB690583	AB690634	AB690664
	CBS 118360	HQ643386	HQ665082	HQ708433	KJ595418
*P. mercuriale*	CBS 122443	AB725882	AB690585	AB690636	AB690666
*P. montanum*	CBS 111349	AB725883	AB690586	AB690637	AB690667
*P. oedochnilum*	CBS 25270	AB690618	AB690592	AB690643	AB690673
	CBS 29237	AB690619	AB690595	AB690646	AB690676
	GUCC5091	AB920534	NA	AB920493	AB920500
*P. ostracodes*	CBS 76873	AY598663	AB690587	AB690638	AB690668
*P. megacarpum*	CBS 112351	HQ643388	HQ665067	HQ708435	AB690665
*P. boreale*	CBS 55188	HQ643372	HQ665261	HQ708419	AB690677
*P. mirpurense*	CBS 124523	KJ831613	KJ831613	KJ831612	NA
*P. vexans*	GUCC0056	AB690626	AB690604	AB690656	AB690686
	GUCC7025	AB690630	AB690608	AB690660	AB690690
	GUCC7010	AB690627	AB690605	AB690657	AB690687
	GUCC7011	AB690628	AB690606	AB690658	AB690688
	GUCC7037	AB690610	AB690581	AB690632	AB690662
	CBS 11980	HQ643400	HQ665090	HQ708447	GU133518
	P133	MT740895	MT729990	MT720669	MT720685
	2D111	AB725880	AB856796	AB856784	AB948193
	ZS01	OM663739	OP159402	OM791301	OM791302
	ZS02	OM663740	OP159403	OM791303	OM791304
	ZS03	OM663741	OP159404	OM791305	OM791306
	HNJC71	PZ022717.1	PZ022833.1	PZ284476	PZ284474
	HNJC90	PZ022718.1	PZ022834.1	PZ284477	PZ284475
*P. nicotianae*	GF101	AB688384	AB688538	AB688265	NA

**Table 3:** Morphometric comparison of sporangia and oogonia between isolates HNJC71 and HNJC90.

Structure	Parameter	HNJC71 (*n* = 100)	HNJC90 (*n* = 100)
Sporangium	Length (μm)	12.62–16.36 (mean = 13.19)	12.91–17.18 (mean = 13.48)
	Width (μm)	8.57–16.45 (mean = 12.41)	8.09–17.23 (mean = 11.57)
	Length × Width (μm)	13.19 × 12.41	13.48 × 11.57
Oogonium	Diameter (μm)	14.88–28.12 (mean = 22.34)	14.23–27.79 (mean = 21.52)
Antheridium	Type	Monoclinous (predominantly), rarely diclinous	Monoclinous (predominantly), rarely diclinous
Oogonial wall	Position	Terminal	Terminal
	Surface	Smooth	Smooth
	Position	Terminal, occasionally intercalary	Terminal, occasionally intercalary

**Table 4:** Quantitative pathogenicity data for isolates HNJC71 and HNJC90 on rubber seedlings.

Parameter	HNJC71	HNJC90
Latent period (dpi)	7	7
Disease incidence at 7 dpi (%)	100	100
Mean lesion length at 7 dpi (mm)	1.54	1.96
Mean lesion length at 14 dpi (mm)	4.68	4.92
Mean lesion length at 21 dpi (mm)	9.35	10.18
Disease severity at 21 dpi (% leaf area affected)	60–85	60–85
Final disease incidence (%)	100	100
Disease incidence at 2 dpi (%)	0	0
Disease incidence at 4 dpi (%)	15.2	17.6
Disease incidence at 7 dpi (%)	73.2	76.7
Disease incidence at 9 dpi (%)	100	100
Final disease incidence (%)	100	100

Note: dpi = days post-inoculation. Data are presented as mean values from three independent biological replicates (*n* = 3), each with three technical replicates. Variability is expressed as mean ± SD (SD values are available upon request). Statistical significance between treatments (HNJC71, HNJC90, and control) at each time point was determined by one-way ANOVA followed by Duncan’s multiple range test (*p* < 0.05).

**Table 5:** Toxicity of six fungicides against the tested strains HNJC71 and HNJC90.

Fungicide	Strain	EC_50_ (μg/mL)	*r* ^2^	Regression Equation	95% Confidence Interval
96% Metalaxyl	HNJC90	0.04	0.91	*y* = 1.11*x* + 1.60	0.03–0.05
	HNJC71	0.06	0.94	*y* = 0.92*x* + 1.17	0.05–0.07
Metalaxyl·propamocarb	HNJC90	0.43	0.98	*y* = 1.65*x* − 0.60	0.40–0.46
	HNJC71	0.44	0.99	*y* = 1.62*x* − 0.61	0.41–0.47
91.7% Fenoconazole	HNJC90	18.64	0.94	*y* =1.15*x* − 1.47	16.29–21.12
	HNJC71	19.50	0.94	*y* = 1.20*x* − 1.54	17.12–22.03
Dimethomorph	HNJC90	134.16	0.95	*y* = 1.69*x* − 3.36	124.34–145.50
	HNJC71	140.60	0.93	*y* = 1.79*x* − 3.93	124.06–161.84

Note: Regression equations were derived from probit-transformed inhibition rates versus log_10_-transformed concentrations (probit-linear model).

## Data Availability

The original contributions presented in this study are included in the article. Further inquiries can be directed to the corresponding author.
